# Supplementary material for: Immune Heterogeneity Between Primary Tumors and Corresponding Metastatic Lesions and Response to Platinum Therapy in Primary Ovarian Cancer
Source: Cancers (Basel). 2019 Aug 26;11(9):1250. doi: 10.3390/cancers11091250 (PMC6769550; doi:10.3390/cancers11091250)
Supplement: Supplementary file 1 [file cancers-11-01250-s001.pdf]

Supplementary materials

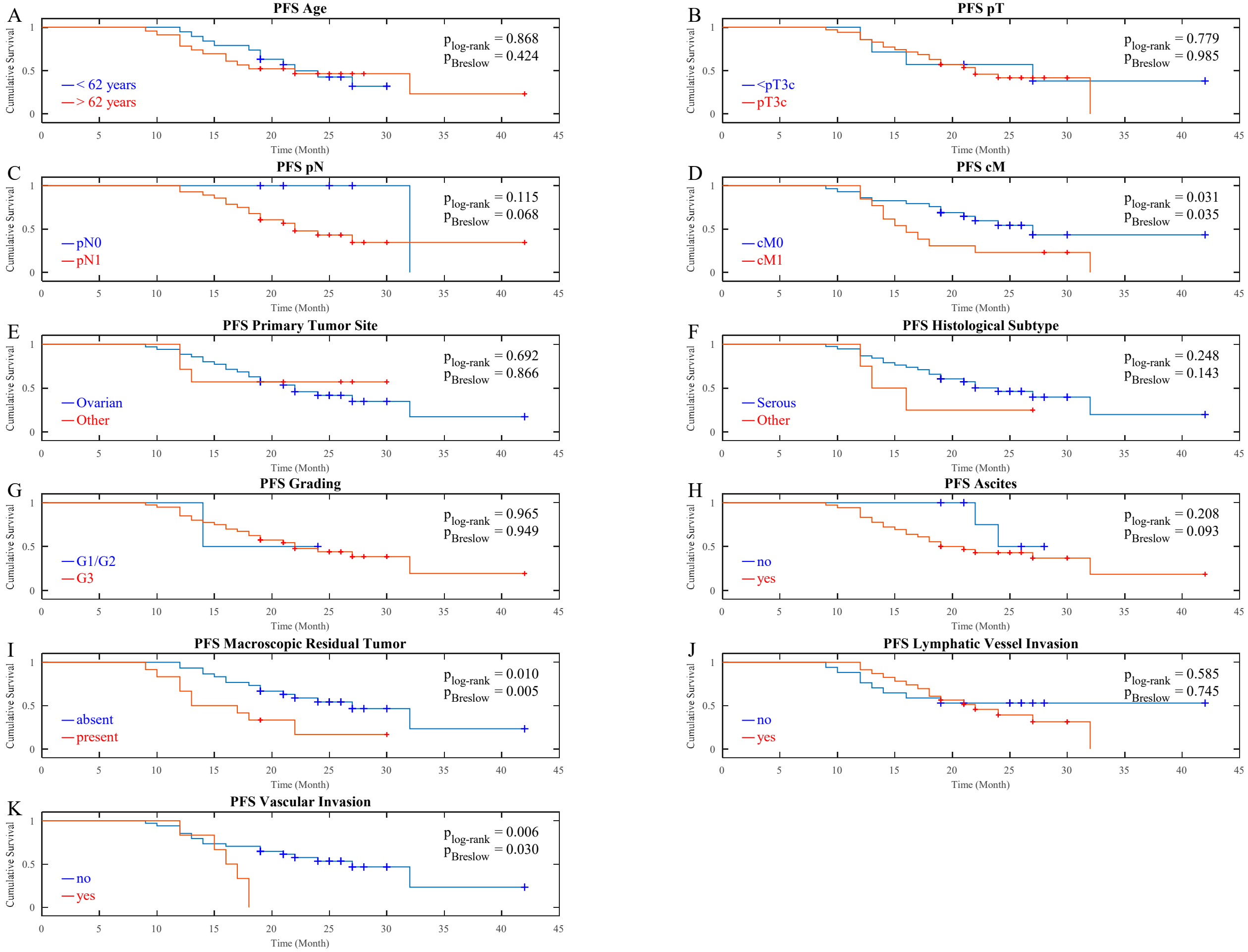

Figure S1. Kaplan-Mayer-Curves for progression free interval (PFS) of different clinicopathological parameters. (A) Age (B) pT (C) pN (D) cM (E) Primary Tumor Site (F) Histological Subtype (G) Grading (H) Ascites (I) Macroscopic Residual Tumor (J) Lymphatic Vessel Invasion (K) Vascular Invasion. p: pathological, c: clinical, T: extent of primary tumor, N: regional lymph node metastasis, M: distant metastasis. *p*-values calculated by log-rank-test and Breslow-test.

Table S1. Clinicopathological factors in relation to platinum-sensitivity.

	n	Platinum-Sensitivity* reduced	full	$p^{\#}$
**Age**	42			0.191
≤ 62 years		4	15	
> 62 years		10	13	
**pT**	42			0.668
< pT3c		3	4	
pT3c		11	24	
**pN**	33			0.559
pN0		0	5	
pN1		7	21	
**cM**	42			**0.015**
cM0		6	23	
cM1		8	5	
**Primary Tumor Site**	42			0.668
Ovarian		11	24	
Other		3	4	
**Histological Subtype**	42			0.1
Serous		11	27	
Other		3	1	
**Grading**	42			1
G1/G2		1	1	
G3		13	27	
**Ascites**	42			0.083
No		0	6	
Yes		14	22	
**Macroscopic Residual Tumor after Surgery**	42			0.067
Absent		7	23	
Present		7	5	
**Lymphatic Vessel Invasion**	40			0.521
No		7	10	
Yes		7	16	
**Vascular Invasion**	40			0.159
No		10	24	
Yes		4	2	

n: number of patients, p: pathological, c: clinical, T: extent of primary tumor, N: regional lymph node metastasis, M: distant metastasis. *Platinum-sensitivity was defined as follows: reduced (relapse ≤12 months after chemotherapy) and full (relapse >12 months after chemotherapy), $^{\#}p$-value calculated by Fisher's exact two-tailed test.

Table S2. Immune cell phenotypes in primary tumor in relation to clinicopathological factors.

	Age				pT				pN				cM				Primary Tumor Site				Histological Subtype				Grading				Ascites				Macroscopic Residual Tumor				Lymphytic Vessel Invasion				Vascular Invasion			
	n	≤ 62	>62	$p^{\#}$	n	<pT3c	pT3c	$p^{\#}$	n	pN0	pN1	$p^{\#}$	n	cM0	cM1	$p^{\#}$	n	Ovarian	Other	$p^{\#}$	n	Serous	Other	$p^{\#}$	n	G1/G2	G3	$p^{\#}$	n	no	yes	$p^{\#}$	n	absent	present	$p^{\#}$	n	no	yes	$p^{\#}$	n	no	yes	$p^{\#}$
**CD45 stromal**	49			0.542	49			0.41	38			0.154	49			**0.042**	49			0.702	49			0.616	49			0.494	49			1	49			1	47			0.528	47			0.244
**Low**		7	7			1	13			0	11			13	1			12	2			12	2			1	13			2	12			10	4			5	9			13	1	
**High**		14	21			7	28			6	21			22	13			27	8			32	3			1	34			6	29			25	10			16	17			25	8	
**CD45 intratumoral**	49			0.779	49			1	38			0.663	49			1	49			1	49			1	49			1	49			**0.006**	49			1	47			0.239	47			0.486
**Low**		11	16			4	23			4	16			19	8			21	6			24	3			1	26			8	19			19	8			14	12			22	4	
**High**		10	12			4	18			2	16			16	6			18	4			20	2			1	21			0	22			16	6			7	14			16	5	
**CD3 stromal**	49			0.144	49			0.438	38			0.682	49			0.222	49			1	49			0.376	49			0.5	49			0.71	49			0.222	47			0.374	47			0.465
**Low**		15	13			6	22			3	19			22	6			22	6			24	4			2	26			4	24			22	6			14	13			23	4	
**High**		6	15			2	19			3	13			13	8			17	4			20	1			0	21			4	17			13	8			7	13			15	5	
**CD3 intratumoral**	49			0.246	49			0.715	38			0.395	49			0.53	49			0.152	49			0.056	49			1	49			1	49			0.207	47			0.076	47			0.263
**Low**		14	13			5	22			4	14			18	9			19	8			22	5			1	26			4	23			17	10			15	11			23	3	
**High**		7	15			3	19			2	18			17	5			20	2			22	0			1	21			4	18			18	4			6	15			15	6	
**CD8 stromal**	49			0.771	49			0.438	38			0.395	49			0.222	49			0.726	49			0.376	49			1	49			0.263	49			0.542	47			1	47			0.465
**Low**		13	15			6	22			2	18			22	6			23	5			24	4			1	27			3	25			21	7			12	15			23	4	
**High**		8	13			2	19			4	14			13	8			16	5			20	1			1	20			5	16			14	7			9	11			15	5	
**CD8 intratumoral**	49			**0.037**	49			0.693	38			1	49			0.527	49			0.726	49			0.143	49			1	49			1	49			0.744	47			0.562	47			0.274
**Low**		17	14			6	25			4	20			21	10			24	7			26	5			1	30			5	26			23	8			14	15			25	4	
**High**		4	14			2	16			2	12			14	4			15	3			18	0			1	17			3	15			12	6			7	11			13	5	
**PD-1 stromal**	49			1	49			0.202	38			0.154	49			1	49			0.702	49			1	49			1	49			1	49			0.294	47			0.528	47			**0.013**
**Low**		15	20			4	31			2	23			25	10			27	8			31	4			2	33			6	29			23	12			16	17			30	3	
**High**		6	8			4	10			4	9			10	4			12	2			13	1			0	14			2	12			12	2			5	9			8	6	
**PD-1 intratumoral**	49			0.76	49			0.411	38			0.392	49			0.501	49			0.464	49			1	49			1	49			1	49			1	47			0.355	47			0.121
**Low**		15	18			4	29			3	22			25	8			25	8			29	4			2	31			6	27			23	10			16	16			28	4	
**High**		6	10			4	12			3	10			10	6			14	2			15	1			0	16			2	14			12	4			5	10			10	5	
**PD-L1 Positivity**	49			0.376	49			1	38			0.37	49			**0.049**	49			1	49			1	49			0.145	49			1	49			1	47			0.775	47			0.278
**No**		10	9			3	16			1	15			17	2			15	4			17	2			2	17			3	16			14	5			9	10			17	2	
**Yes**		11	19			5	25			5	17			18	12			24	6			27	3			0	30			5	25			21	9			12	16			21	7	

n: number of patients, p: pathological, c: clinical, T: extent of primary tumor, N: regional lymph node metastasis, M: distant metastasis, PD-1: programmed cell-death protein 1, PD-L1: programmed cell-death ligand 1. $^{\#}$ *p*-value calculated by Fisher's exact two-tailed test.

Table S3. Comparison of the immune cell phenotypes between primary tumor and corresponding lesions.

	n PT > OM	n PT = OM	n PT < OM	$p^{\#}$	n PT > PE	n PT = PE	n PT < PE	$p^{\#}$
**CD45 stromal**	2	9	12	0.007	3	9	3	0.739
**CD45 intratumoral**	3	18	2	0.655	4	10	1	0.48
**CD3 stromal**	7	0	16	0.005	4	1	10	0.331
**CD3 intratumoral**	8	1	14	0.221	8	0	7	0.82
**CD8 stromal**	7	0	16	0.012	8	0	7	0.82
**CD8 intratumoral**	7	1	15	0.131	4	2	9	0.124
**PD-1 stromal**	7	1	15	0.013	8	1	6	0.95
**PD-1 intratumoral**	9	7	7	0.569	10	3	2	0.054
**PD-L1 Expression**	4	15	4	0.944	1	10	4	0.078

n: number of analyzed tumor samples, PT: Primary tumor, OM: Omental lesion, PE: Peritoneal lesion, PD-1: programmed cell-death protein 1, PD-L1: programmed cell-death ligand 1.
p-value calculated by Wilcoxon signed-rank test.

Table S4. Ratios of immune cell phenotypes between primary tumor and omental lesion in relation to clinicopathological factors.

	Age				pT				pN				cM				Primary Tumor Site			
	n	≤ 62	>62	$p^{\#}$	n	<pT3c	pT3c	$p^{\#}$	n	pN0	pN1	$p^{\#}$	n	cM0	cM1	$p^{\#}$	n	Ovarian	Other	$p^{\#}$
**CD45 stromal**	23			0.193	23			1	19			**0.018**	23			0.590	23			0.069
**OM ≤ PT**		2	9			1	10			4	4			9	2			6	5	
**OM > PT**		6	6			1	11			0	11			11	1			11	1	
**CD45 intratumoral**	23			1	23			1	19			1	23			0.249	23			1
**OM ≤ PT**		7	14			2	19			4	13			19	2			15	6	
**OM > PT**		1	1			0	2			0	2			1	1			2	0	
**CD3 stromal**	23				23				19			**0.037**	23				23			
**OM ≤ PT**		1	6			0	7			3	2			6	1			4	3	
**OM > PT**		7	9			2	14			1	13			14	2			13	3	
**CD3 intratumoral**	23			0.086	23			0.142	19			0.603	23			1	23			0.643
**OM ≤ PT**		1	8			2	7			2	5			8	1			6	3	
**OM > PT**		7	7			0	14			2	10			12	2			11	3	
**CD8 stromal**	23			1	23			1	19			0.272	23			0.526	23			0.318
**OM ≤ PT**		2	5			0	7			2	3			7	0			4	3	
**OM > PT**		6	10			2	14			2	12			13	3			13	3	
**CD8 intratumoral**	23			0.176	23			0.111	19			0.557	23			1	23			0.621
**OM ≤ PT**		1	7			2	6			2	4			7	1			5	3	
**OM > PT**		7	8			0	15			2	11			13	2			12	3	
**PD-1 stromal**	23			0.657	23			1	19			0.071	23			1	23			0.621
**OM ≤ PT**		2	6			1	7			3	3			7	1			5	3	
**OM > PT**		6	9			1	14			1	12			13	2			12	3	
**PD-1 intratumoral**	23			1	23			1	19			0.255	23			0.526	23			0.621
**OM ≤ PT**		6	10			2	14			4	9			13	3			11	5	
**OM > PT**		2	5			0	7			0	6			7	0			6	1	
**PD-L1 Positivity**	23			0.257	23			1	19				23			0.453	23			0.539
**OM ≤ PT**		8	11			2	17			3	13	0.530		17	2			13	6	
**OM > PT**		0	4			0	4			1	2			3	1			4	0	

	Histological Subtype				Grading				Ascites				Macroscopic Residual Tumor				Lymphytic Vessel Invasion				Vascular Invasion			
	n	Serous	Other	$p^{\#}$	n	G1/G2	G3	$p^{\#}$	n	no	yes	$p^{\#}$	n	absent	present	$p^{\#}$	n	no	yes	$p^{\#}$	n	no	yes	$p^{\#}$
**CD45 stromal**	23			1	23			0.478	23			0.590	23			1	22			0.395	22			0.476
**OM ≤ PT**		10	1			0	11			2	9			8	3			7	4			11	0	
**OM > PT**		10	2			2	10			1	11			8	4			4	7			9	2	
**CD45 intratumoral**	23			1	23			0.170	23			1	23			1	22			1	22			1
**OM ≤ PT**		18	3			1	20			3	18			14	7			10	11			19	2	
**OM > PT**		2	0			1	1			0	2			2	0			1	0			1	0	
**CD3 stromal**	23			1	23			1	23			1	23			1	22			1	22			1
**OM ≤ PT**		6	1			0	7			1	6			5	2			4	3			6	1	
**OM > PT**		14	2			2	14			2	14			11	5			7	8			14	1	
**CD3 intratumoral**	23			1	23			0.502	23			0.538	23			0.176	22			0.659	22			0.515
**OM ≤ PT**		8	1			0	9			2	7			8	1			5	3			8	0	
**OM > PT**		12	2			2	12			1	13			8	6			6	8			12	2	
**CD8 stromal**	23			1	23			1	23			0.209	23			0.626	22			1	22			1
**OM ≤ PT**		6	1			0	7			2	5			4	3			4	3			6	1	
**OM > PT**		14	2			2	14			1	15			12	4			7	8			14	1	
**CD8 intratumoral**	23			1	23			1	23			0.269	23			0.345	22			1	22			0.515
**OM ≤ PT**		7	1			1	7			2	6			7	1			4	4			8	0	
**OM > PT**		13	2			1	14			1	14			9	6			7	7			12	2	
**PD-1 stromal**	23			1	23			0.526	23			1	23			0.657	22			1	22			0.121
**OM ≤ PT**		7	1			0	8			1	7			5	3			4	4			6	2	
**OM > PT**		13	2			2	13			2	13			11	4			7	7			14	0	
**PD-1 intratumoral**	23			1	23			0.526	23			1	23			1	22			1	22			1
**OM ≤ PT**		14	2			1	15			2	14			11	5			8	7			13	2	
**OM > PT**		6	1			1	6			1	6			5	2			3	4			7	0	
**PD-L1 Positivity**	23			1	23			0.324	23			1	23			1	22			0.586	22			1
**OM ≤ PT**		16	3			1	18			3	16			13	6			10	8			16	2	
**OM > PT**		4	0			1	3			0	4			3	1			1	3			4	0	

n: number of patients, PT: Primary tumor, OM: Omental lesion, p: pathological, c: clinical, T: extent of primary tumor, N: regional lymph node metastasis, M: distant metastasis, PD-1: programmed cell-death protein 1, PD-L1: programmed cell-death ligand 1. [#] *p*-value calculated by Fisher's exact two-tailed test.

Table S5. Ratios of immune cell phenotypes between primary tumor and peritoneal lesion in relation to clinicopathological factors.

	Age				pT				pN				cM				Primary Tumor Site				Histological Subtype				Grading				Ascites				Macroscopic Residual Tumor				Lymphytic Vessel Invasion				Vascular Invasion			
	n	≤ 62	>62	$p^{\#}$	n	<pT3c	pT3c	$p^{\#}$	n	pN0	pN1	$p^{\#}$	n	cM0	cM1	$p^{\#}$	n	Ovarian	Other	$p^{\#}$	n	Serous	Other	$p^{\#}$	n	G1/G2	G3	$p^{\#}$	n	no	yes	$p^{\#}$	n	absent	present	$p^{\#}$	n	no	yes	$p^{\#}$	n	no	yes	$p^{\#}$
**CD45 stromal**	15			1	15			0.371	11			/	15			1	15			0.516	15			/	15			/	15			0.242	15			1	14			0.176	14			1
**PE ≤ PT**		6	6			1	11				10			7	5			10	2			12					12			3	9			9	3			2	9			9	2	
**PE > PT**		1	2			1	2				1			2	1			2	1			3					3			2	1			2	1			2	1			2	1	
**CD45 intratumoral**	15			0.467	15			1	11			/	15			1	15			1	15			/	15			/	15			0.333	15			1	14			1	14			1
**PE ≤ PT**		6	8			2	12				10			8	6			11	3			14					14			4	10			10	4			4	9			10	3	
**PE > PT**		1	0			0	1				1			1	0			1	0			1					1			1	0			1	0			0	1			1	0	
**CD3 stromal**	15			0.282	15			0.524	11			/	15			0.329	15			1	15			/	15			/	15			1	15			1	14			0.520	14			1
**PE ≤ PT**		1	4			0	5				4			2	3			4	1			5					5			2	3			4	1			2	2			3	1	
**PE > PT**		6	4			2	8				7			7	3			8	2			10					10			3	7			7	3			2	8			8	2	
**CD3 intratumoral**	15			0.619	15			0.467	11			/	15			0.119	15			1	15				15				15			1	15			0.569	14			0.559	14			1
**PE ≤ PT**		3	5			2	6				4			3	5			6	2			8		/			8	/		3	5			5	3			3	4			5	2	
**PE > PT**		4	3			0	7				7			6	1			6	1			7					7			2	5			6	1			1	6			6	1	
**CD8 stromal**	15			0.619	15			0.2	11			/	15			0.608	15			0.2	15			/	15			/	15			1	15			0.569	14			0.559	14			1
**PE ≤ PT**		3	5			0	8				6			4	4			5	3			8					8			3	5			5	3			3	4			5	2	
**PE > PT**		4	3			2	5				5			5	2			7	0			7					7			2	5			6	1			1	6			6	1	
**CD8 intratumoral**	15			0.119	15			0.486	11			/	15			0.622	15			0.525	15			/	15			/	15			0.329	15			1	14			0.245	14			1
**PE ≤ PT**		1	5			0	6				4			3	3			4	2			6					6			3	3			4	2			3	3			5	1	
**PE > PT**		6	3			2	7				7			6	3			8	1			9					9			2	7			7	2			1	7			6	2	
**PD-1 stromal**	15			0.119	15			0.486	11			/	15			0.622	15			0.229	15			/	15			/	15			0.580	15			0.604	14			0.085	14			0.538
**PE ≤ PT**		6	3			2	7				6			6	3			6	3			9					9			4	5			6	3			4	4			7	1	
**PE > PT**		1	5			0	6				5			3	3			6	0			6					6			1	5			5	1			0	6			4	2	
**PD-1 intratumoral**	15			0.200	15			1	11			/	15			0.486	15			0.371	15			/	15			/	15			1	15			1	14			0.505	14			1
**PE ≤ PT**		5	8			2	11				9			7	6			11	2			13					13			4	9			9	4			3	9			9	3	
**PE > PT**		2	0			0	2				2			2	0			1	1			2					2			1	1			2	0			1	1			2	0	
**PD-L1 Positivity**	15			1	15			1	11			/	15			0.604	15			1	15			/	15			/	15			0.231	15			1	14			0.251	14			0.505
**PE ≤ PT**		5	6			2	9				8			6	5			9	2			11					11			5	6			8	3			4	6			7	3	
**PE > PT**		2	2			0	4				3			3	1			3	1			4					4			0	4			3	1			0	4			4	0	

n: number of patients, PT: Primary tumor, PE: Peritoneal lesion, p: pathological, c: clinical, T: extent of primary tumor, N: regional lymph node metastasis, M: distant metastasis, PD-1: programmed cell-death protein 1, PD-L1: programmed cell-death ligand 1. # *p*-value calculated by Fisher's exact two-tailed test.

Table S6. Description of primary tumor site and corresponding lesions per patient.

Patient Number	Primary Tumor Site—According to Pathological Report	Location of Corresponding Lesions	
		**Omentum**	**Peritoneum**
5064	Ovary	Greater Omentum	
5085	Ovary		Peritoneum*
5188	Ovary	Greater Omentum	
4747	Ovary	Greater Omentum	Sigma
4757	Ovary		Peritoneum*
4976	Ovary		Peritoneum*
5074	Ovary	Greater Omentum	
5146	Ovary	Greater Omentum	
5196	Ovary	Greater Omentum	
5199	Ovary	Greater Omentum	
5243	Ovary	Greater Omentum	
5306	Ovary	Greater Omentum	Peritoneum*
4751	Fallopian Tube	Greater Omentum	
4754	Ovary		Peritoneum*
5072	Ovary	Greater Omentum	Sigma
5082	Fallopian Tube		Sigma
5097	Ovary	Greater Omentum	
5170	Fallopian Tube	Greater Omentum	
5216	Fallopian Tube	Greater Omentum	
5264	Ovary	Greater Omentum	Diaphragm
5266	Fallopian Tube	Greater Omentum	
5277	Peritoneum	Greater Omentum	Uterus
5281	Peritoneum	Greater Omentum	Diaphragm
4843	Ovary	Greater Omentum	
4940	Ovary		Peritoneum*
5075	Ovary		Liver Capsule
5242	Ovary	Greater Omentum	
5070	Ovary	Greater Omentum	
5088	Ovary	Greater Omentum	
5103	Ovary		Sigma
5121	Ovary	Greater Omentum	
5137	Ovary		Peritoneum*

* not further specified.

Table 7. Description of rating method.

Rating	Individual Cells	Cluster
0	none	none
1	scattered	none
2	a few	none
3	a few	small, locally restricted
4	many	small and big, locally restricted
5	many	big, confluent
